# Molecular and Anatomical Strengthening of “Winner” Climbing Fiber Synapses in Developing Mouse Purkinje Cells

**DOI:** 10.1523/JNEUROSCI.2156-24.2025

**Published:** 2025-02-27

**Authors:** Asako Nitta, Miwako Yamasaki, Taisuke Miyazaki, Kohtarou Konno, Haruto Yoshimura, Masahiko Watanabe

**Affiliations:** ^1^Department of Anatomy, Graduate School of Medicine, Hokkaido University, Sapporo 060-8638, Japan; ^2^Department of Anesthesiology, School of Medicine, Sapporo Medical University, Sapporo 060-8543, Japan; ^3^Department of Anatomy, Faculty of Medicine, Hokkaido University, Sapporo 060-8638, Japan; ^4^Department of Functioning and Disability, Faculty of Health Sciences, Hokkaido University, Sapporo 060-8638, Japan; ^5^School of Medicine, Hokkaido University, Sapporo 060-8638, Japan

**Keywords:** AMPA receptor, climbing fiber, dendritic spine, Purkinje cell, synapse, synapse elimination

## Abstract

Neural circuits are refined by strengthening frequently used or advantaged synapses while eliminating redundant connections. In neonatal mice, cerebellar Purkinje cells (PCs) are initially innervated by multiple climbing fibers (CFs) of similar strength. By postnatal day 7 (P7), one CF, the “winner,” is selectively strengthened and begins dendritic translocation by P9, while both “winner” and “loser” CFs temporarily maintain somatic synapses. Although the functional differentiation of CF inputs is well understood, their structural differentiation is less clear. In this study, we examined “winner” CF synapses in dendrites and both “winner” and “loser” synapses in the soma using serial electron microscopy and immunohistochemistry in C57BL/6 mice. We found that “winner” CF synapses, both in the soma and dendrites, developed more complex pre- and postsynaptic structures than “loser” CFs, with an expanded area of postsynaptic density. Additionally, “winner” CF synapses expressed significantly higher levels of AMPA-type glutamate receptors. Notably, only dendritic “winner” synapses showed increased levels of Rab3-interacting molecule RIM, a key presynaptic regulator of neurotransmitter release. These findings reveal the molecular and structural features that enable “winner” CFs to reinforce their synaptic strength and innervation, allowing them to outcompete other inputs during early development.

## Significance Statement

The neural circuit is refined by selectively strengthening specific synapses while eliminating redundant connections. In neonates, cerebellar Purkinje cell somata are innervated by multiple climbing fibers (CFs) with similar strength. Subsequently, a single CF is strengthened as the “winner,” establishing a stable connection by translocating to dendrites. While the functional differentiation of CFs has been well-characterized, their structural differentiation remains largely unclear. Through serial electron microscopy and immunohistochemistry, we reveal that “winner” CF synapses elaborated synaptic structures with elevated AMPA receptor expression and presynaptic Rab-interacting molecule RIM. Thus, translocated “winner” CF synapses undergo molecular and anatomical strengthening, securing an irreversible competitive advantage over somatic CF synapses. The results provide a developmental basis for better understanding synaptic circuit refinement.

## Introduction

The proper function of the nervous system relies on the precise formation of neural circuits. In neonates, neurons initially form redundant and supernumerary synaptic connections. Early postnatal development refines the immature circuits by selectively strengthening and stabilizing essential synapses while weakening and eliminating excess connections (Purves and Lichtman, 1980; Katz and Shatz, 1996; Lichtman and Colman, 2000; Hensch, 2004). This process, known as synapse elimination, plays a critical role in transforming immature circuits into mature, functional neural circuits in an activity-dependent manner across various regions of the central and peripheral nervous systems (Lichtman and Colman, 2000; Kano and Hashimoto, 2009). The climbing fiber (CF) to Purkinje cell (PC) synapse in the cerebellum provides an excellent model for investigating the cellular and molecular mechanisms of circuit refinement in the developing central nervous system (Crepel, 1982; Lohof et al., 1996; Hashimoto and Kano, 2005; Hashimoto et al., 2009b).

Multiple CFs innervating neonatal PC somata exhibit similar synaptic strengths (Hashimoto and Kano, 2003) and structures (Sugihara, 2005). Shortly after birth, CFs project numerous creeping terminals to the PC layer without forming aggregations around specific PC somata (Chedotal and Sotelo, 1992; Sugihara, 2005). Between the postnatal day 4 (P4) and P7, multiple CFs from different neuronal origins form pericellular nests (Mason et al., 1990; Sugihara, 2005; Hashimoto et al., 2009a), from which a single CF becomes functionally strengthened through activity-dependent mechanisms (Hashimoto and Kano, 2003; Bosman et al., 2008; Ohtsuki and Hirano, 2008; Kawamura et al., 2013). From P9 on, the strengthened CF (“winner” CF) expands its innervation territory and begins “climbing” along the newly formed apical dendrite (Hashimoto et al., 2009a; Carrillo et al., 2013; Ichikawa et al., 2016), while both “winner” and “loser” CFs temporarily retain somatic synapses. Specifically, the pruning of somatic CF synapses starting around P12 critically depends on the formation and activity of parallel fiber (PF) synapses, another excitatory input to PCs (Hashimoto et al., 2009b), and CF mono-innervation is established in most PCs by approximately P21. Thus, the CF synapse refinement is marked by distinct stages of initial synapse formation, strengthening, and elimination. The mechanisms underlying the functional strengthening of “winner” CFs and the elimination of redundant “loser” CFs are relatively well understood (Kano et al., 2018). One hypothesis suggests that dendritic translocation confers an irreversible competitive advantage to the “winner” CFs (Carrillo et al., 2013). Nevertheless, the structural and molecular differentiation associated with the functional strengthening of “winner” CFs remains largely unexplored.

In this study, we employed immunohistochemistry and serial electron microscopy (EM) to investigate the molecular and anatomical differentiation of multiple CF inputs, with particular emphasis on comparing CF synapses formed by “winner” and “loser” inputs, as well as the distinction between dendritic and somatic synapses formed by “winner” CFs, before, during, and after the peak of dendritic translocation. We found that, compared to “loser” CF synapses, “winner” CF synapses developed more complex and elaborate synaptic structures, expanded the postsynaptic density (PSD), and expressed higher levels of AMPA-type glutamate receptors (AMPARs). Furthermore, “winner” CF synapses translocated to dendrites exhibited significant and specific up-regulation of Rab3-interacting molecule RIM, a key regulator of neurotransmitter release at the presynaptic active zone. Our data reveal structural and molecular correlates underlying the selective molecular and anatomical strengthening of single “winner” CFs, which subsequently expand their innervation territory and establish mono-innervation to PCs.

## Materials and Methods

### Animals

Animal experiments were performed according to the guidelines laid down by the Hokkaido University animal experiment committee (Protocol #19-0111, #24-0054). Mice were housed at 22–24°C with a standard 12 h light-dark cycle, ad libitum water access, and a standard chow. C57BL/6 mice were analyzed at postnatal day (P)7, 9, 10, 12, 14, 15, and 21.

### Antibodies

Primary antibodies against the following molecules were used: Alexa488, calbindin, pan-AMPAR, PSD95, RIM1/2, TARPγ2 and VGluT2. Information on the molecule, antigen sequence, host species, specificity, reference, NCBI GenBank accession number, and RRID are summarized in Table 1. The dilution of antibodies in each experiment was described in sections in Materials and Methods.

**Table 1.** Details of primary antibodies used

Molecule	Sequence Provider	#NCBI	Host	Specificity	RRID	Reference
Alexa488	Invitrogen (A-11094)		Rb	IB	AB_221544	
calbindin	abcam (ab82812)		Mo	IB	AB_1658451	
panAMPAR	727–745 aa	X57497	Gp	IB/HEK	AB_2571610	Fukaya et al., 2006
PSD95	1–62 aa	D50621	Gp	IB	AB_2920798	Fukaya and Watanabe, 2000
RIM1/2	SY (140217)		Rt	KO	AB_2924949	
TARPγ2	302–318 aa	AF077739	Rb	KO/IB	AB_2571844	Yamazaki et al., 2010
VGluT2	559–582 aa	BC038375	Go	IB	AB_2571619	Miyazaki et al., 2003

aa, amino acid residues; Go, goat polyclonal antibody; Gp, guinea pig polyclonal antibody; IB, immunoblot with brain homogenates; IB/HEK, immunoblot with HEK293 cells transfected with relevant mouse molecule; KO, lack of immunolabeling in knockout brains; Mo, mouse monoclonal antibody; Rb, rabbit polyclonal antibody; Rt, rat monoclonal antibody. Antibody dilution for each experiment is described in the individual sections of the Materials and Methods.

### Anterograde tracer labeling

Under deep anesthesia with approximately 2% isoflurane (Pfizer), a glass pipette (G-1.2; Narishige) filled with 2–3 μl of a 10% dextran solution conjugated with Alexa Fluor-488 (DA488; Invitrogen) in PBS was stereotaxically inserted into the inferior olive using a dorsal approach (Miyazaki and Watanabe, 2011). 80 nl of DA488 was delivered over 3 min via a syringe pump (Micro4, World Precision Instruments). Dextran injections were performed at P5, with animals sacrificed at P7–P9; at P10, with sacrifices at P12, P14, and P15; and at P15, with sacrifice at P21.

### Immunofluorescence

Mice were deeply anesthetized with an overdose of pentobarbital (100 mg/kg, i.p.), and then perfused transcardially with 5 ml of saline, followed by 60 ml of glyoxal fixative solution (9% (v/v) glyoxal (Merck), 8% (v/v) acetic acid, pH 4.0) (Richter et al., 2018; Konno et al., 2023) and postfixed overnight at 4°C. Subsequently, brains were immersed in a 30% sucrose solution/0.1 M PB (pH 7.2) for cryoprotection. Parasagittal sections (50-μm-thick) were prepared from the cerebellar vermis using a cryostat (CM1860, Leica Microsystems) and immunostained using the free-floating method in glass test tubes. Phosphate-buffered saline (PBS, pH 7.4) containing 0.1% Triton X-100 (PBS-T) was used as the incubation and washing buffer. Sections were blocked with 10% normal donkey serum (Jackson ImmunoResearch) for 20 min, then incubated overnight at room temperature with a mixture of primary antibodies (1 µg/ml each). Following incubation, sections were washed and then incubated for 2 h at room temperature with DyLight405, Alexa488, Cy3, and Alexa647-labeled species-specific secondary antibodies (Jackson ImmunoResearch; Thermo Fisher Scientific). After washing, sections were mounted on APS-coated glass slides (Matsunami, Osaka, Japan), air-dried, and coverslipped using ProLong Glass (Thermo Fisher Scientific).

### Immunofluorescence image acquisition and analysis

**Figure 1.**
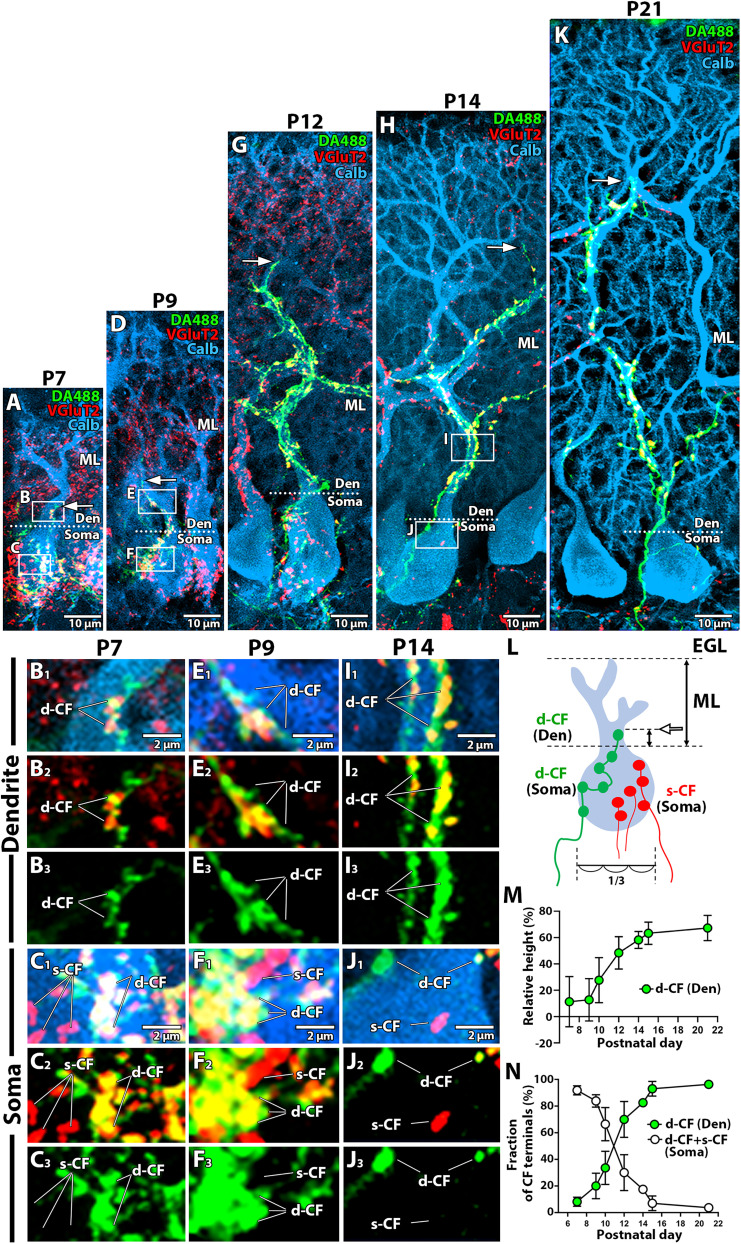
Developmental profile of CF innervation from P7 to P21. ***A–K***, Triple fluorescent labeling for anterograde tracer dextran Alexa488 (DA488, green), type2 vesicular glutamate transporter (VGluT2, red), calbindin (calb, blue) at P7 (***A–C***), P9 (***D–F***), P12 (***G***), P14 (***H–J***) and P21 (***K***). Arrows indicate the most distal tip of the DA488-labeled CF terminal. Boxed regions in ***A***, ***D***, and ***H*** are enlarged and separated in each channel in ***B***, ***C***, ***E***, ***F***, ***I***, and ***J***. CF terminals are classified as double-labeled for DA488 and VGluT2 (d-CF) or single-labeled for VGluT2 only (s-CF). ***L***, Schematic of Purkinje cells (PC) innervated with d-CF (green) and s-CF (red). During development, the external granule cell layer (EGL) lies above the molecular layer (ML). ML thickness is measured as the vertical distance from the most distal tip of PC dendrites to the apex of the PC soma, defined as the point where the soma narrows to less than 1/3 of its diameter. The schematic below the PC illustrates one-third of the diameter of the PC soma. The vertical distance from the most distal CF terminal (arrow) to the top of the soma was also measured, and the relative height of CF terminals was calculated as their ratio. ***M***, Developmental changes in the relative height of the most distal d-CF terminals. ***N***, Developmental changes in the fraction of the CF terminal area of d-CF on dendrites (green) and d-CF and s-CF in the soma (white). The sample size used for measurement is *n* = 9–13 CFs in each stage from 3–7 *z*-stacked images across 2–3 mice (***L***); *n* = 5 PCs in each stage from 5 *z*-stacked images across 2–3 mice (***M,N***). Data are presented as mean ± SD.

Photographs were taken with a confocal laser scanning microscope (FV1200, Evident) equipped with 405, 473, 559, and 647 nm diode lasers and a UPlanXApo (60/1.42 Oil) objective lens. Fluorescence cross-talk was minimized by narrowing the emission wavelength using a spectral slit and by employing the sequential laser scanning mode. Images were *z*-stacked at 0.5 μm intervals over a 2–5 μm thickness to follow the trajectory of DA488-labeled CFs. For measurement, the point where the soma narrows to less than 1/3 of its diameter was defined as the apex of the PC soma. The molecular layer (ML) thickness was measured as the vertical distance from the apex of the soma to the distal tip of the dendrites (Fig. 1*L*). The vertical distance from the apex of the soma to the most distal CF terminal was also measured, and the relative height of CF terminals was calculated as their ratio. For quantification of the fraction of CF terminals (Fig. 1*N* and relevant analysis), DA488 and/or VGluT2 immunopositive terminals were semi-automatically detected using the “Inclusive Threshold” and “Create Regions Around Objects” functions in MetaMorph (Molecular Devices). Images for Figures 5, 7, 8, were taken with a digital zoom × 3. For quantitative analysis, images were separated into individual channels and converted to 8-bit grayscale. Measurements were performed using MetaMorph. For Figures 5–8, the regions of interest (ROI) for measurements were defined manually around the VGluT2 intensity. The average signal intensity (in arbitrary units, A.U.) was measured in each ROI.

### Pre-embedding immunoelectron microscopy and EM analysis

Under deep anesthesia with pentobarbital (100 mg/kg, i.p.), a mouse at P10 or P12 was transcardially perfused with 4% PFA/0.1 M PB (pH 7.2), supplemented with 0.1% glutaraldehyde, for 10 min. The brain was then postfixed for 2 h in the same fixative, and parasagittal sections (50-μm-thick) were prepared from the cerebellar vermis using a vibratome (VT1000S, Leica Microsystems). PBS (pH 7.4) containing 0.1% Tween 20 was used as an incubation and washing buffer. Sections were blocked with 10% normal goat serum (Nichirei Bioscience Corporation, Tokyo, Japan) for 20 min, and incubated overnight at room temperature with either rabbit anti Alexa488 antibody (1 μg/ml; for P10 samples) or guinea pig anti-VGluT2 antibody (1 μg/ml; for P12 samples). After washing, sections were incubated with 1.4 nm colloidal gold-conjugated rabbit or guinea pig IgG antibody (1 : 100; Nanoprobes, Stony Brook, USA) for 4 h at room temperature. Sections were washed with HEPES Buffer (50 mM HEPES, 200 mM sucrose, 5 N sodium hydroxide) (pH 8.0), and then incubated with silver enhancement reagent (AURION R-Gent SE-EM; AURION, Wageningen, Netherlands) for 30 min. Sections were fixed with 1% osmium tetroxide solution on ice for 15 min, and then stained with 2% uranyl acetate for 15 min, dehydrated in a graded ethanol series and n-butyl glycidyl ether, embedded in epoxy resin, and polymerized at 60°C for 48 h. Ultrathin sections in the plane parallel to the section surface were prepared with an ultramicrotome (UCT7, Leica Microsystems). The average section thickness was assumed to be 80 nm, based on the minimal folds method (Small, 1968), which is in close agreement with the estimates (∼80 nm) obtained by interference coloration of the sections floating on water in the boat of the diamond knife (Meek, 1976). Serial sections were mounted on indium-tin-oxide-coated glass slides (IT5-111-50, NANOCS, Boston, MA) and successively stained with 2% uranyl acetate and lead citrate. After washing, colloidal graphite (Ted Pella Inc, Redding, CA) was pasted on the glass slides to surround the ribbons. Sequential images (210 and 233 for P12 and P10 samples, respectively) were acquired using a scanning electron microscope (SEM) with a backscattered electron beam detector at an accelerating voltage of 1.0 kV and a magnification of ×10,000 (2,560 pixels × 1,920 pixels, for P12 samples) or ×5,000 (5,120 pixels × 3,840 pixels, for P10 samples) (SU8240, Hitachi High Technologies, Tokyo, Japan).

For 3D reconstruction, SEM images with voxel dimensions of 5 nm × 5 nm × 80 nm (xyz) were aligned using TrakEM2 (Cardona et al., 2012) (plug-in for NIH ImageJ, FIJI), loaded into Image Pro 10 (for P12 samples; Media Cybernetics, Rockville, MD) or Dragonfly (version 2022.2.0.1409) (for P10 samples; Object Research Systems, Montreal, Canada), and the structures of interest were manually segmented by delineating their boundary contours to create 3D surface renderings. Synaptic profiles were sampled only if clearly identified and fully included within serially sectioned tissue volumes. Spines were visually identified as membranous protrusions extending from the soma or dendrite, characterized by their contacts with axon terminals, the presence of endoplasmic reticulum, and the absence of mitochondria. The boundary between the spine neck and the parent dendritic shaft or soma was determined by drawing a consistent tangential line across the base of the spine neck. In rare instances where branched spines were observed, they were further divided into individual spines at their shared neck isthmus. For P12 samples, total spine volume was calculated by multiplying the traced cytoplasmic area by the section thickness. For P10 samples, total spine volume was calculated by creating a 3D contour mesh (threshold = 20; sampling rate at 1) and verified using voxel-based measurement. For both samples, total PSD areas were calculated by multiplying the summed PSD lengths (measured on each ultrathin section and summed from all sections through each synapse) by the section thickness.

### Statistical analysis

No data were excluded from the analysis. Data are presented as mean ± SD in graphs and text, and as box plots showing the median, interquartile range (IQR), and whiskers representing the 10th–90th percentiles (where *n* = number of analyzed neuronal profiles, cells, or ROIs, unless otherwise noted). Graphing and statistical tests were performed using GraphPad Prism 10 (GraphPad Software). Normality was determined with D’Agostino & Pearson, Anderson-Darling, Shapiro-Wilk, or one sample Kolmogorov-Smirnov tests. In all cases, the assumption of normality is not met for all groups, statistics were performed using non-parametric tests. For three or more group comparisons, we conducted the Kruskal-Wallis test. If significant differences were detected, Dunn’s test assessed post-hoc multiple comparisons. Pearson correlation and simple linear regression analyses were performed to investigate the relationship between the two variables. The difference in linear regression slope was determined using analysis of covariance (ANCOVA). All figures present statistical significance as *p < 0.05, **p < 0.01, ***p < 0.001, ****p < 0.0001.

## Results

### Anatomical differentiation of multiple CF inputs into “winner” and “loser” CFs

We first investigated the anatomical differentiation of multiple CF inputs into “winner” and “loser” CFs during a developmental period from P7 to P21 (Fig. 1). To avoid interlobular differences in development (Altman and Bayer, 1997; Sugihara, 2005), we limited our analysis to PCs in the lobule 4 + 5 of the cerebellar vermis. We visualized CF innervation in PCs by triple fluorescent labeling for calbindin (PC marker, blue), VGluT2 (CF terminal marker, red), and anterograde tracer dextran Alexa 488 (DA488, green) injected into the inferior olive. To distinguish potential “winner” CFs and “loser” CFs of different neuronal origins, we sampled PCs innervated by DA488-labeled CFs that formed the highest terminal among VGluT2-labeled CF terminals. To ensure certainty, only DA488-labeled CF axons that were continuously and unambiguously traceable from the soma to the dendrites were analyzed. Although PF terminals transiently express VGluT2 during early postnatal development (Miyazaki et al., 2003), the two inputs can be readily distinguished: CF terminals appeared as large perisomatic or peridendritic puncta intense for VGluT2, whereas PF terminals were discerned as small weak neuropil puncta in the superficial molecular layer.

At P7, CF terminals, which were double-labeled for DA488 and VGluT2 (d-CF, yellow or white puncta) or single-labeled for VGluT2 (s-CF, red puncta), were distributed around the soma in most PCs (Fig. 1*A*,*C*). Occasionally, a few d-CF terminals were reached in the very basal part of the primary dendrite (Fig. 1*A*,*B*). At P9, a fraction of d-CF terminals translocated to the basal part of the primary dendrite in many PCs (Fig. 1*D*,*E*), while numerous d-CF and s-CF terminals remained in the soma (Fig. 1*D*,*F*). At P12 and P14, d-CF terminals extended robustly along the developing proximal dendrites (Fig. 1*G–I*). In contrast, somatic d-CF and s-CF terminals were substantially reduced from P12 to P14 (Fig. 1*J*). By P21, the anatomical pattern of CF mono-innervation, as being characterized by exclusive innervation of the proximal PC dendrites by a single d-CF, was commonly established (Fig. 1*K*).

The following quantitative analyses confirmed the above observations. Dendritic translocation was evaluated by calculating the relative CF height, which was defined as the ratio of two vertical distances: the distance from the apex of PC soma to the most distal d-CF punctum relative to the thickness of the molecular layer (ML) (Fig. 1*L*). The relative CF height increased gradually from P9 and reached a plateau at P15 (Fig. 1*M*). Next, we examined the composition of CF terminals in the soma and dendrites (Fig. 1*N*). However, accurate counting of CF terminal numbers was often challenging due to the irregular shape and small size of VGluT2-labeled CF terminals at immature stages. To overcome this issue, we measured the summed area of d-CF and s-CF terminals around the soma and along the dendritic shafts for evaluation. Until P9, somatic CF terminals (s-CF and d-CF) constituted more than 80% of the total CF terminals (Fig. 1*N*, white dots). This proportion drastically decreased thereafter and almost disappeared by P21. Conversely, dendritic d-CF terminals increased from P7, surpassed somatic CF terminals at P12, and reached a plateau at P15 (Fig. 1*N*, green dots). This rise in dendritic d-CF terminals closely paralleled the increase in relative CF height (Fig. 1*M*). On the contrary, the proportion of somatic CF terminals progressively decreased from P10 or P12 to P21 (Fig. 1*N*).

For further analyses, we classified the postnatal period into three stages: before (P7–P9), during (P10–P12), and after (P14–P15) the peak stage of dendritic translocation. Although most CF terminals remain at the soma, the distal fraction of CF terminals had already translocated to dendrites in P7–P9 (Fig. 1*A*,*B*,*D*,*E*), suggesting that these CFs represent the substantial “winner”. Consequently, we categorized CF synapses into three classes based on their origin and location: “winner” synapses on dendrites, “winner” synapses in the soma, and “loser” synapses in the soma.

### Structural differentiation of CF synapses during the peak of dendritic translocation

We next investigated ultrastructural differences among three synapse classes at P10 and P12, i.e., during the peak of dendritic translocation.

**Figure 2.**
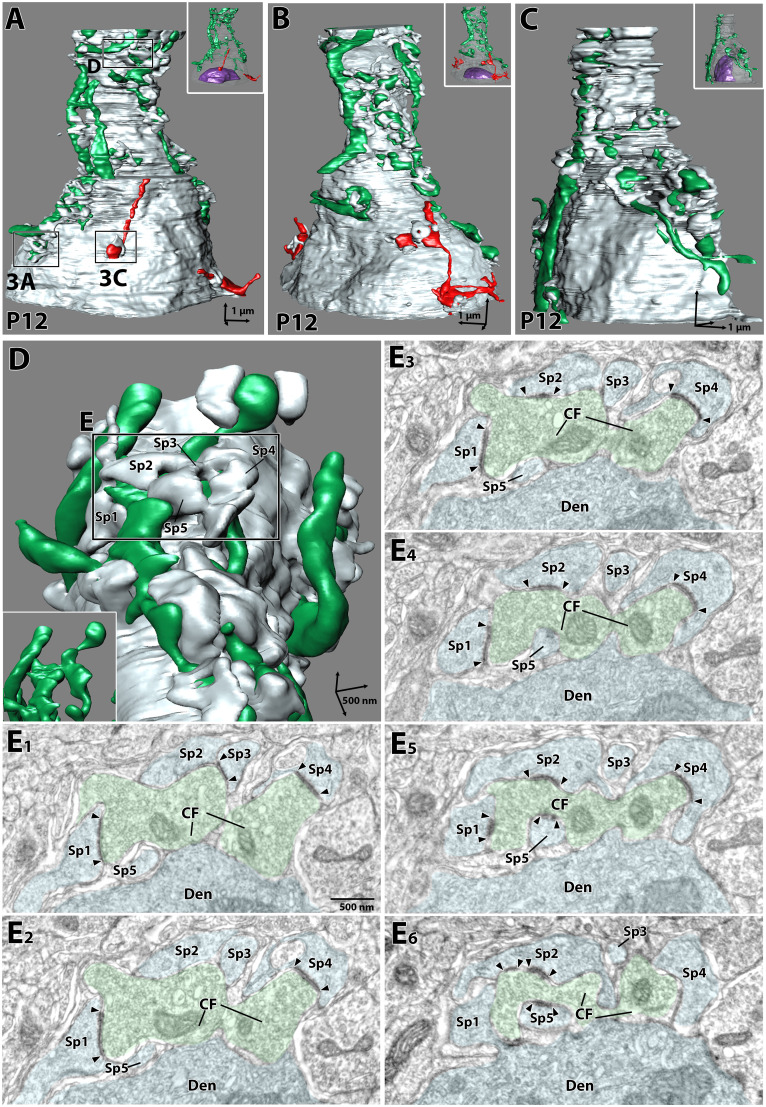
Partial EM reconstruction of CF-PC wiring at P12: Preembedding immunogold EM for VGluT2. ***A–C***, Partial reconstruction of somato-dendritic border regions from three PCs at P12. The “winner” and “loser or unclassified (L/UC)” CFs are shown in green and red, respectively. Boxed regions in (***A***) are enlarged in (***D***) and in Figure 3*A*,*C*. The PC nucleus (purple) is shown in each inset. ***D***, The enlarged image of basal dendrite from the boxed region in (***A***) reveals multiple dendritic spines encapsulating the “winner” CF fiber. Often, a part of the CF bouton (inset) is surrounded by dendritic spines, making it invisible from the front view. ***E***, Six consecutive EM images corresponding to the boxed region in (***D***) show that five spines (Sp1–5) contact a CF terminal. PC and CF profiles are pseudocolored in pale blue and green, respectively.

First, we performed serial EM analysis of the P12 cerebellum labeled for VGluT2 using pre-embedding silver-enhanced immunogold. Due to limited antibody penetration, VGluT2 particles were observed only on the surface, although CF terminals remained traceable. Partial reconstructions were generated from three PCs, each spanning 16.8 μm covering the somato-dendritic border (Fig. 2*A–C*). By following VGluT2-labeled axons across consecutive sections, we were able to track distinct CF branches. Among these, CF branches forming synapses on dendrites were designated as “winner” CFs (Fig. 2*A–C*, green). At P12, they formed axo-spinous synapses on both dendrites and the soma (Fig. 2*A–C*). Furthermore, additional innervation by distinct CF branches was observed in two of the three PCs (Fig. 2*A*,*B*). These branches in the soma lacked a clear connection to the “winner” CF branches, suggesting that they could originate from “loser” CFs of distinct origin or be disconnected from or branch from “winner” axons outside the sampling area. Therefore, we refer to them as “loser/unclassified” (“L/UC”) CFs (Fig. 2*A*,*B*, red).

**Figure 3.**
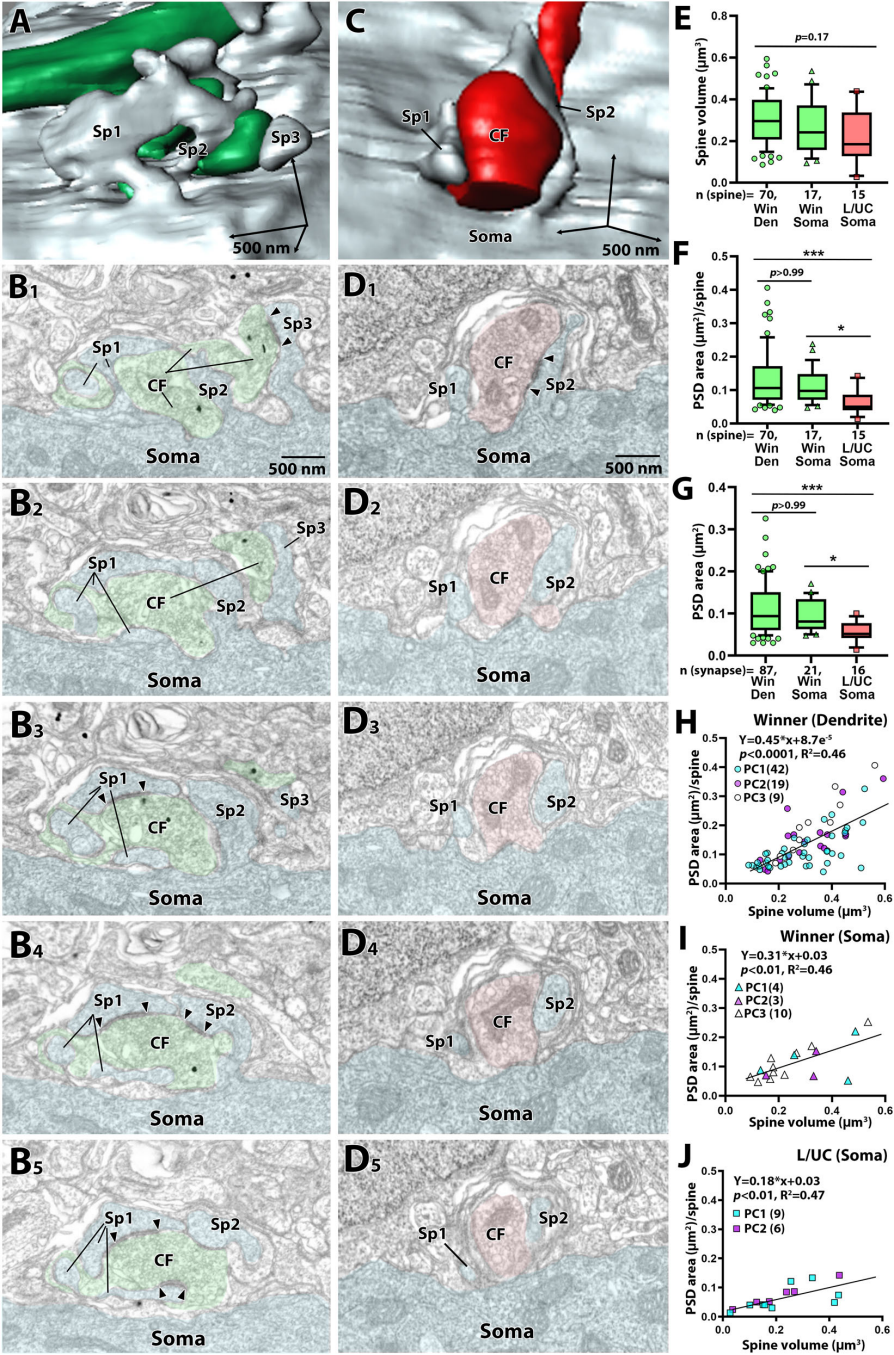
Partial EM reconstruction of CF synapses at P12 using preembedding immunogold for VGluT2 labeling. ***A***, An enlarged image of a “winner” CF from a boxed region in Figure 2*A*. Multiple somatic spines enwrap the “winner” CF. ***B***, Five consecutive EM images corresponding to (***A***) show that three spines (Sp1–3) contact a “winner” CF terminal. PC and CF profiles are pseudocolored in pale blue and green, respectively. ***C***, An enlarged image of the “L/UC” CF from a boxed region in Figure 2*A*. Two somatic spines contact a “L/UC” CF terminal. ***D***, Five consecutive EM images corresponding to (***C***) show that two spines (Sp1 and Sp2) contact a CF terminal, but only Sp2 makes a synapse. PC and CF profiles are pseudocolored in pale blue and red, respectively. Summary box plots showing comparisons in the volume of individual spines (***E***), the PSD area per spine (***F***), and individual PSD area (***G***). The whiskers are drawn down to the 10th percentile and up to the 90th. Data points below and above the whiskers are drawn as individual symbols. The sample size used for statistical testing are indicated below the graph. Data are obtained from 3 PCs. The *p* values are shown at the top of each graph (*p < 0.05, ***p < 0.001): Kruskal-Wallis test (***E***) and Kruskal-Wallis test and post hoc Dunn’s multiple comparisons test (***F, G***). ***H–J***, Scatterplot showing the relationship between the PSD area (ordinate) and spine volume (abscissa) at “winner” CF synapses on dendrites (H), “winner” CF synapses in the soma (***I***), and “loser” CF synapses in the soma (***J***). Data from PC1 (Fig. 2*A*), PC2 (Fig. 2*B*) and PC3 (Fig. 2*C*) are indicated with cyan, magenta, and white, respectively. Data are obtained from the same datasets in (***E–G***). Lines and equations represent the fit from Pearson’s linear regression, with *R*^2^ and *p*-values displayed on the graph.

“Winner” CF terminals innervating dendrites were enlarged and lobulated, while multiple dendritic spines were elongated and enwrapped to contact CF terminals (Fig. 2*D*). For example, a CF terminal segment shown as consecutive views in Figure 2*E* were contacted by five spines, and each spine formed asymmetric synapses, as evidenced by the presence of thickened PSD. This CF terminal formed six synaptic contacts with a summed PSD area of 0.57 μm^2^. “Winner” and “L/UC” CFs also formed synapses with somatic spines (Figs. 2*A*, 3*A–D*). Compared to “L/UC” CF synapses in the soma (Fig. 3*C*,*D*), “winner” CF synapses in the soma tended to exhibit terminal lobulation and spine elongation and enwrapping, similar to “winner” CF synapses in dendrites (Fig. 3*A*,*B*). In contrast, these features were much less clear for “L/UC” CF synapses in the soma, with the simple shape of terminal boutons and short spines (Fig. 3*C*,*D*). Most conspicuously, the size of PSD at individual synaptic contact was clearly smaller at somatic synapses of “L/UC” CFs than those of “winner” CFs in the soma and dendrites (Fig. 3*B*,*D*). A “winner” CF terminal in Figure 3*A* formed four somatic synapses with a total PSD area of 0.31 μm^2^, while a “L/UC” CF terminal in Figure 3*C* formed two somatic synapses, though only one is shown, with a total PSD area of only 0.04 μm^2^.

**Figure 4.**
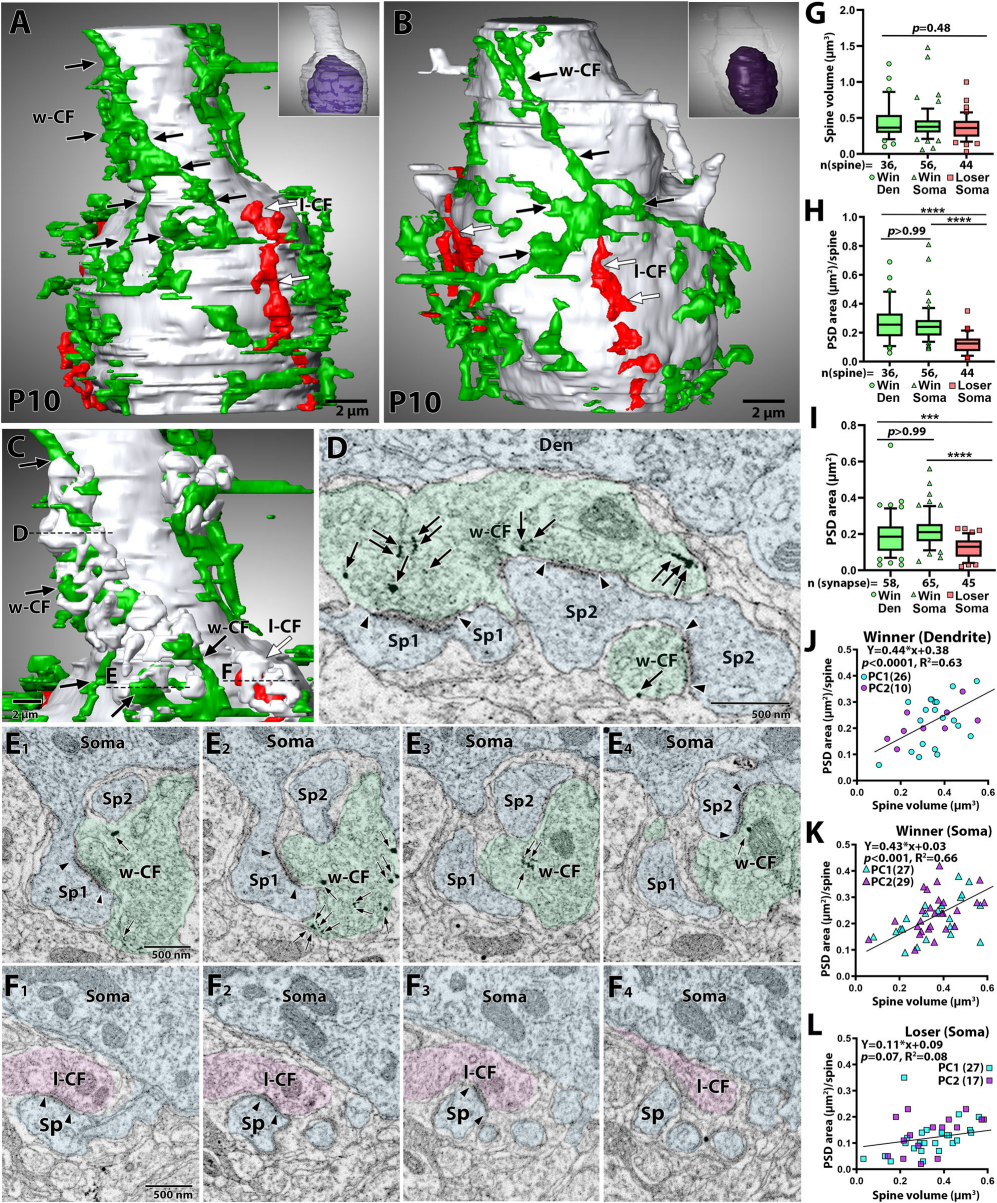
Partial EM reconstruction of CF-PC wiring at P10: Preembedding immunogold for CF tracer labeling. ***A, B***, Partial reconstruction of somato-dendritic border regions from two PCs at P10. DA488-labeled CF is “winner” CF (w-CF) and “loser” CF (l-CF) are shown in green and red, respectively. Continuous branches of “winner” and “lose” CFs are indicated by black and white arrows, respectively. The PC nucleus (purple) is shown in each inset. For clarity, dendritic spines are not shown. ***C***, The enlarged image of basal dendrite from (***A***) reveals multiple dendritic spines encapsulating “winner” and “loser” CFs. EM images indicated with the dashed lines are shown in (***D–F***). ***D***, An EM image show that three spines (Sp1–3) contact a CF terminal labeled with metal particles for DA488 (arrows). PC and CF profiles are pseudocolored in pale blue and green, respectively. ***E***, Four consecutive EM images around the dashed line in (***C***) show somatic “winner” CF synapses. Multiple somatic spines enwrap the “winner” CF. ***F***, Four consecutive EM images corresponding to the dashed line in (***C***) show “loser” synapses in the soma. ***G–I*,** Summary box plots showing comparisons in the volume of individual spines (***G***), the PSD area per spine (***H***), and individual PSD area (***I***). The whiskers are drawn down to the 10th percentile and up to the 90th. Data points below and above the whiskers are drawn as individual symbols. The sample size used for statistical testing are indicated below the graph. The *p* values are shown at the top of each graph (***p < 0.001, ****p < 0.0001): Kruskal-Wallis test (***G***) and Kruskal-Wallis test and post hoc Dunn’s multiple comparisons test (***H, I***). ***J–L***, Scatterplot showing the relationship between the PSD area (ordinate) and spine volume (abscissa) at “winner” CF synapses on dendrites (***J***), “winner” CF synapses in the soma (***K***), and “loser” CF synapses in the soma (***L***). Data from PC1 (***A***) and PC2 (***B***) are indicated with cyan and magenta, respectively. Data are obtained from the same datasets in (***G–I***). Lines and equations represent the fit from Pearson’s linear regression, with *R*^2^ and *p*-values displayed on the graph.

Quantitative measurements from the three PCs revealed that the spine volume (Fig. 3*E*) was comparable among the three classes of CF synapses (Kruskal-Wallis test, *p* = 0.17). Similarly, the number of synapses per dendritic spine did not differ significantly across the three classes of CF synapses (“winner” dendrite: 1.35 ± 0.68, “winner” soma: 1.38 ± 0.51, “L/UC” soma, 1.17 ± 0.41; mean ± SD; Kruskal-Wallis test, *p* = 0.63; from the same dataset shown in Fig. 3*E*,*F*). The total PSD area per dendritic spine and individual PSD area were significantly larger for “winner” CF synapses in the soma and dendrites compared to “L/UC” CF synapses in the soma (Fig. 3*F*,*G*; Kruskal-Wallis test followed by Dunn’s multiple comparisons tests, *p* < 0.001 “winner” dendrite vs “L/UC” soma; *p* < 0.05 “winner” soma vs “L/UC” soma). When plotting the spine volume against the PSD area per spine, a positive correlation was found for all three classes (Fig. 3*H–J*). However, compared to the “winner” synapses in the soma and dendrites, the linear regression slope for the “L/UC” synapses in the soma was shallower (ANCOVA, “winner” dendrite vs “L/UC” soma, *p* < 0.0001 F = 15.98, DFn = 1, DFd = 76; “winner” soma vs “L/UC” soma, *p* < 0.0001 F = 19.20, DFn = 1, DFd = 96), suggesting a weaker or minimal relationship.Next, to directly compare ultrastructural differences between “winner” and “loser” CF synapses, we applied serial EM with pre-embedding immunogold labeling to DA488-labeled CFs at P10 (Fig. 4). We sampled DA488-labeled “winner” CFs, which were traced continuously from the soma to dendrites, and generated 3D partial reconstructions from two PCs spanning 18.6 μm to cover the somato-dendritic border (Fig. 4*A*,*B*). Both PCs exhibited mixed innervation by DA488-labeled “winner” CFs and unlabeled “loser” CFs, clearly demonstrating their different neuronal origins (Fig. 4*A*,*B*). Furthermore, based on our earlier observations (Ichikawa et al., 2011), basket cell synapses on somatic spines are less common before P12 and typically form synapses simultaneously on the soma and spines. In contrast, “loser” CF synapses predominantly target somatic spines without associated somatic synapses. This distinct synaptic pattern allowed us to distinguish “loser” CF synapses from potential basket cell inputs.

Consistent with the observations at P12 (Figs. 2 and 3), “winner” CF synapses in dendrites (Fig. 4*C*,*D*) and the soma (Fig. 4*E*) exhibited complex features, such as lobulated CF terminals and spines (Fig. 4*C–E*). In contrast, these structural features were markedly less pronounced in “loser” CF synapses in the soma (Fig. 4*F*). Quantitative analysis from the two PCs showed that spine volume was comparable across the three classes of CF synapses (Fig. 4*G*; Kruskal-Wallis test, *p* = 0.48). However, the total PSD area per spine (Fig. 4*H*) and individual PSD area (Fig. 4*I*) was significantly larger for “winner” CF synapses on dendrites and the soma compared to “loser” CF synapses in the soma (Kruskal-Wallis test with Dunn's multiple comparisons, *p* < 0.0001 for “winner” soma vs “loser” soma; *p* < 0.05 for “winner” dendrite vs “loser” soma).

When spine volume was plotted against PSD area per spine, a positive correlation emerged for “winner” synapses on dendrites and the soma (Fig. 4*J*,*K*), but not for “loser” synapses in the soma (Fig. 4*L*). These findings demonstrate that “winner” CF synapses on dendrites and the soma develop more elaborate structures and larger PSDs compared to “loser” CF synapses in the soma.

### Synaptic molecule expression at three classes of CF synapses

Then, we compared the expression level of pre- and post-synaptic molecules among the three classes of CF synapses at three postnatal stages. To this end, we utilized the glyoxal fixation protocol, which enables efficient detection of synaptic molecules (Konno et al., 2023).

#### PSD95

**Figure 5.**
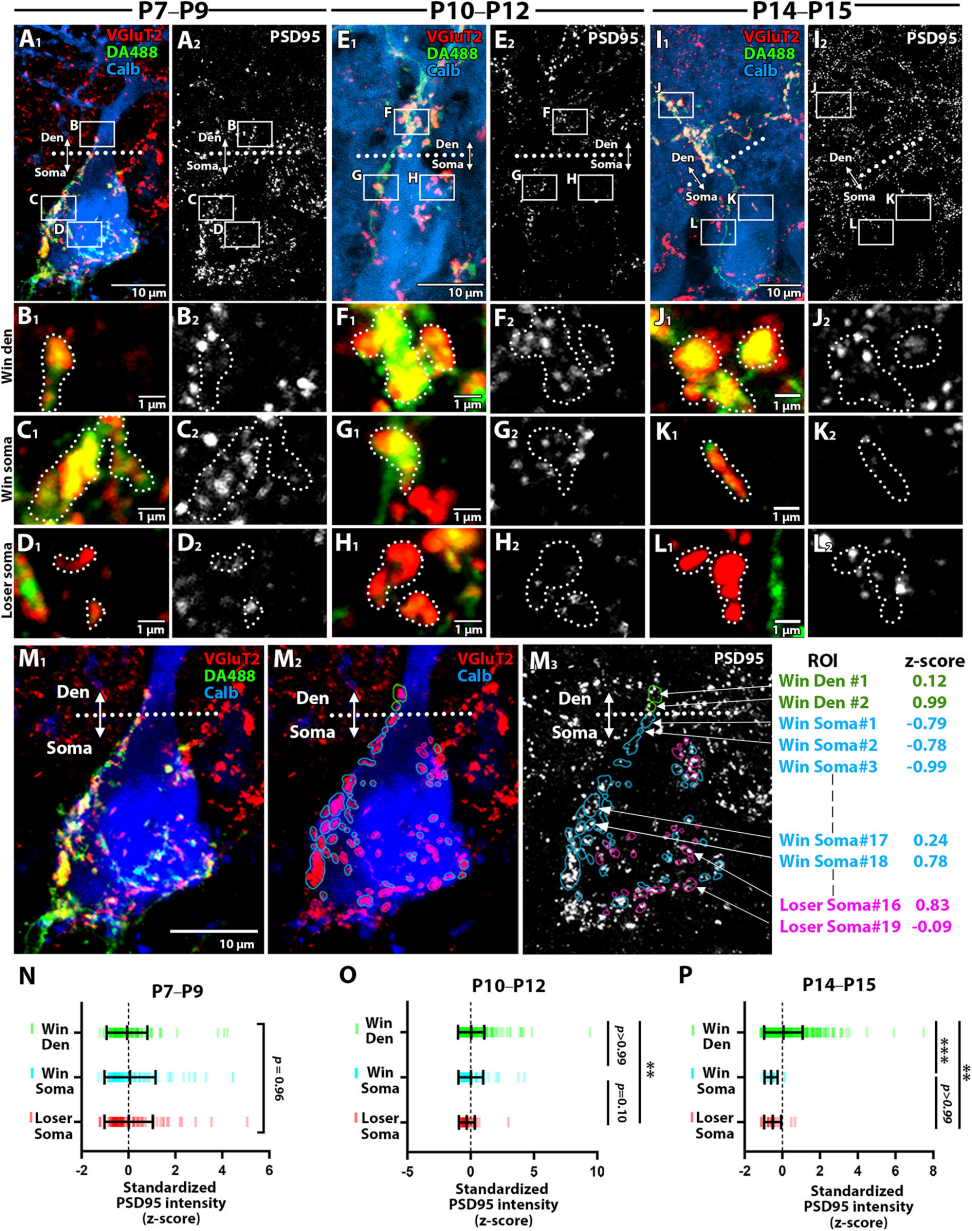
PSD95 expression across three classes of CF synapses at three developmental stages. ***A–L***, Quadplex fluorescent labeling for DA488 (green), VGluT2 (red), calbindin (blue), and PSD95 (white) to compare the expression level of PSD95 across the three types of CF synapses at P7–P9 (left), P10–P12 (middle), and P14–P15 (right). Dotted lines in ***A***, ***E***, and ***I*** represent the somato-dendritic border. Boxed regions in ***A***, ***E***, and ***I*** are enlarged in ***B–D***, ***F–H***, and ***J–L***, respectively. Dotted lines in ***B–D***, ***F–H***, and ***J–L*** indicate the contour of CF terminals. ***M***, Regions of interest (ROIs) around VGluT2-labeled CF terminals were defined, and the signal intensity of each ROI was measured. In M_2_ and M_3_, ROIs for “winner” CF terminals on dendrites and the soma were delineated with green and blue lines, respectively, while “loser” CF terminals were outlined in magenta. To minimize variation in signal intensities between images, the intensity values of each ROI within each PC were standardized as described in the Results. Sample images were taken from (***A***). ***N–P***, Summary plots comparing the standardized signal intensity of PSD95 in each class of CF synapses. Dashes represent the standardized signal in individual CF ROIs. The sample size used for statistical testing of “winner” dendrite, “winner” soma, and “loser” soma is *n* = 197, 118, and 100 CF ROIs from 8 PCs across 3 mice (P7–P9); *n* = 530, 90, and 28 CF ROIs from 5 PCs across 2 mice (P10–P12); *n* = 1,063, 26, and 19 CF ROIs from 5 PCs across 2 mice (P14–P15). Mean ± SD is shown for each plot. **p < 0.01, ***p < 0.001, Kruskal-Wallis test and post hoc Dunn’s multiple comparisons.

By quadruple fluorescent labeling for calbindin (blue), VGluT2 (red), DA488 (green), and PSD95 (white), we first compared PSD95, an essential scaffold protein of glutamatergic postsynapse (Fig. 5). At P7–P9, when somatic spines are the primary targets of CF innervation, perisomatic PSD95 labeling predominated, with only a fraction of terminals observed on the primary dendrite (Fig. 5*A–D*). In the soma and dendrites, multiple PSD95 puncta were associated with individual d-CF and s-CF terminals, suggesting the formation of multiple synaptic sites in each terminal (Fig. 5*B–D*). At P10–P12 and P14–P15, CF terminals associated with dendritic PSD95 puncta progressively increased with a reciprocal decrease in the soma (Fig. 5*E–L*). “Winner” and “loser” CF terminals in the soma retained association with PSD95 puncta, but the signal intensity was gradually decreased (Fig. 5*G*,*H*,*K*,*L*).

In this and subsequent expression analyses, we compared expression levels across three synapse classes in different PCs. Regions of interest (ROIs) were defined around VGluT2-labeled CF terminals, and the signal intensity of each ROI was measured. To minimize variation in signal intensities between images, we standardized the intensity values of each ROI in each PC by calculating the *z*-score (Fig. 5*M*) (Harris et al., 2022), which represents how many standard deviations each value deviates from the mean. The *z*-score was calculated as *z* = (*X* - *μ*)/*σ*, where *X* is the signal intensity of the individual ROI, μ is the mean intensity of all ROIs in each PC, and σ is the standard deviation of all ROIs in each PC. We did not observe significant differences in standardized PSD95 intensity across the three synapse types at P7–P9 (Fig. 5*N*; Kruskal-Wallis test, *p* = 0.96). At P10–P12, PSD95 labeling was highest at “winner” CF synapses on dendrites than “loser” CF synapses in the soma (Fig. 5*O*; Kruskal-Wallis test followed by Dunn's multiple comparisons tests, *p* < 0. 01), but there were no significant differences between the dendritic and somatic “winner” CF synases (Kruskal-Wallis test, *p* > 0.99) or between the somatic “winner” and “loser” CF synapses (Kruskal-Wallis test, *p* = 0.10). At P14–P15, the trend was similar except that PSD95 expression in the “winner” somatic synapses was significantly lower than those in the dendritic synapses (Fig. 5*P*; Kruskal-Wallis test followed by Dunn’s multiple comparisons test, *p* < 0.001 for “winner” dendrite vs “winner” soma). Therefore, PSD95 expression is comparable across three synapses before the peak of translocation, but is up-regulated at “winner” CF synapses on dendrites during and after the peak of dendritic translocation.

#### AMPAR

**Figure 6.**
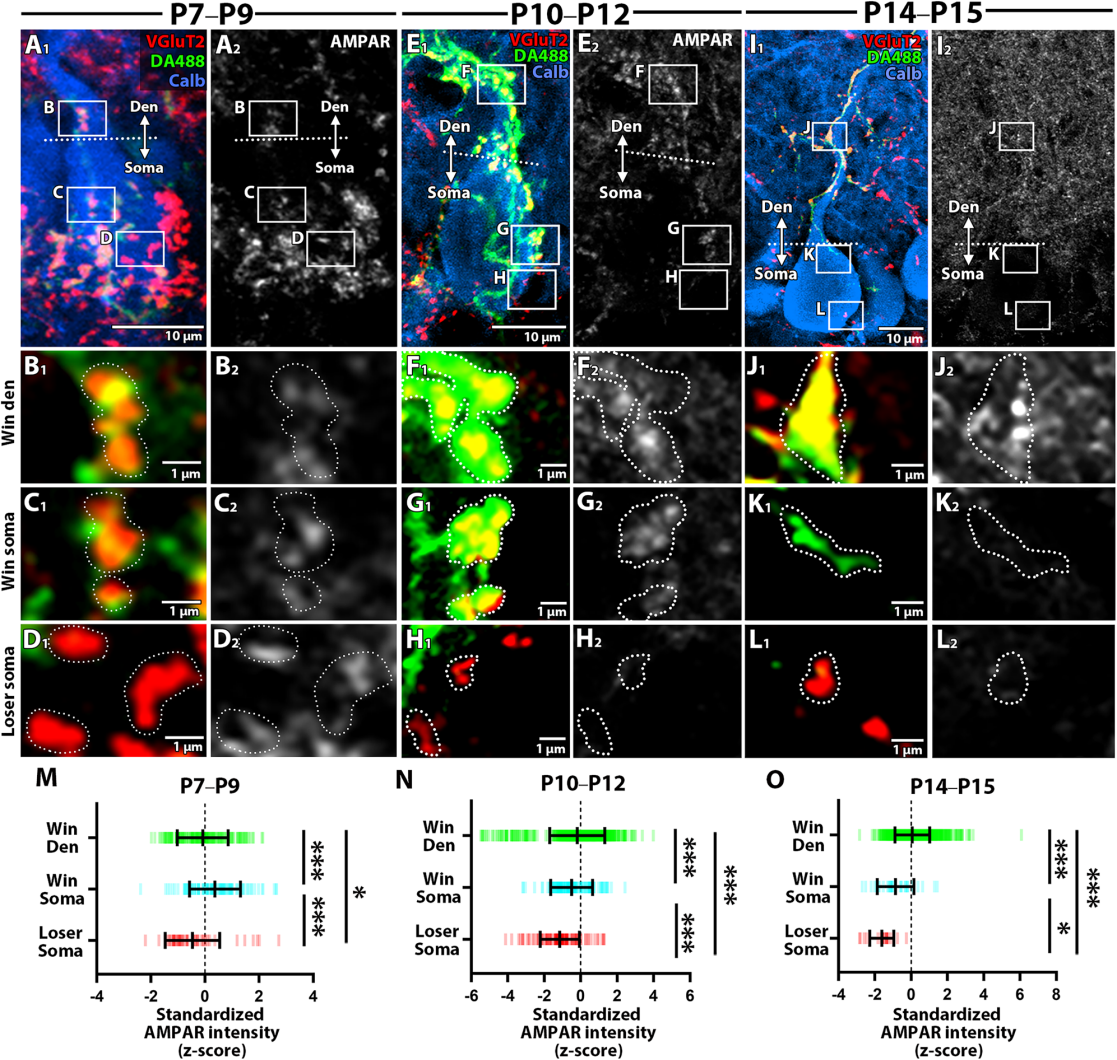
AMPAR expression across three classes of CF synapses at three developmental stages. Quadplex fluorescent labeling for DA488 (green), VGluT2 (red), calbindin (blue), and AMPAR (white) to compare the expression level of AMPARs. See the explanation in Figure 5. The sample size used for statistical testing of “winner” dendrite, “winner” soma, and “loser” soma is *n* = 167, 102, and 51 CF ROIs from 8 PCs across 3 mice (P7–P9); *n* = 743, 136, and 112 CF ROIs from 10 PCs across 5 mice (P10–P12); *n* = 930, 30, and 21 CF ROIs from 8 PCs across 5 mice (P14–P15). Mean ± SD is shown for each plot. *p < 0.05, ***p < 0.001, Kruskal-Wallis test and post hoc Dunn’s multiple comparisons.

We next examined AMPAR expression, a key determinant of the synaptic strength in the postsynapse. Using a pan-AMPAR antibody raised against a sequence common to all four GluA subunits (Fukaya et al., 2006), we conducted quadruple fluorescent labeling for calbindin (blue), VGluT2 (red), DA488 (green), and AMPARs (white) (Fig. 6). At P7–P9, all classes of CF terminals showed, in general, low AMPAR labeling (Fig. 6*B–D*). At P10–P12, AMPAR labeling was intensified at “winner” CF synapses in the soma and dendrites (Fig. 6*E–G*), whereas it was relatively lowered at “loser” CF synapses in the soma (Fig. 6*E*,*H*). At P14–P15 (Fig. 6*I–L*), “winner” CF synapses in dendrites were further intensified, whereas those in the soma were lowered (Fig. 6*J*). By comparison of standardized signal intensity, AMPAR labeling at P7–P9 was highest at “winner” CF synapses in the soma and lowest at “loser” CF synapses in the soma (Fig. 6*M*; Kruskal-Wallis test followed by Dunn’s multiple comparisons tests, *p* < 0.001 for “winner” soma vs “loser” soma and “winner” soma vs “winner” dendrite; *p* < 0.05 for “winner” dendrite vs “loser” soma). At P10–P12, AMPAR labeling was highest at “winner” CF synapses in dendrites and lowest at “loser” CF synapses in the soma (Fig. 6*N*; Kruskal-Wallis test followed by Dunn’s multiple comparisons tests, *p* < 0.001 for all comparisons). This trend became more evident at P14–P15 (Fig. 6*O*; Kruskal-Wallis test followed by Dunn’s multiple comparisons test, *p* < 0.001 for “winner” dendrite vs “winner” soma and “winner” dendrite vs “loser” soma; *p* < 0.05 for “winner” soma vs “loser” soma). Therefore, AMPAR expression is up-regulated at “winner” CF synapses in both the soma and dendrites before and during the peak of dendritic translocation and predominated at “winner” CF synapses in dendrites after the peak of dendritic translocation.

**Figure 7.**
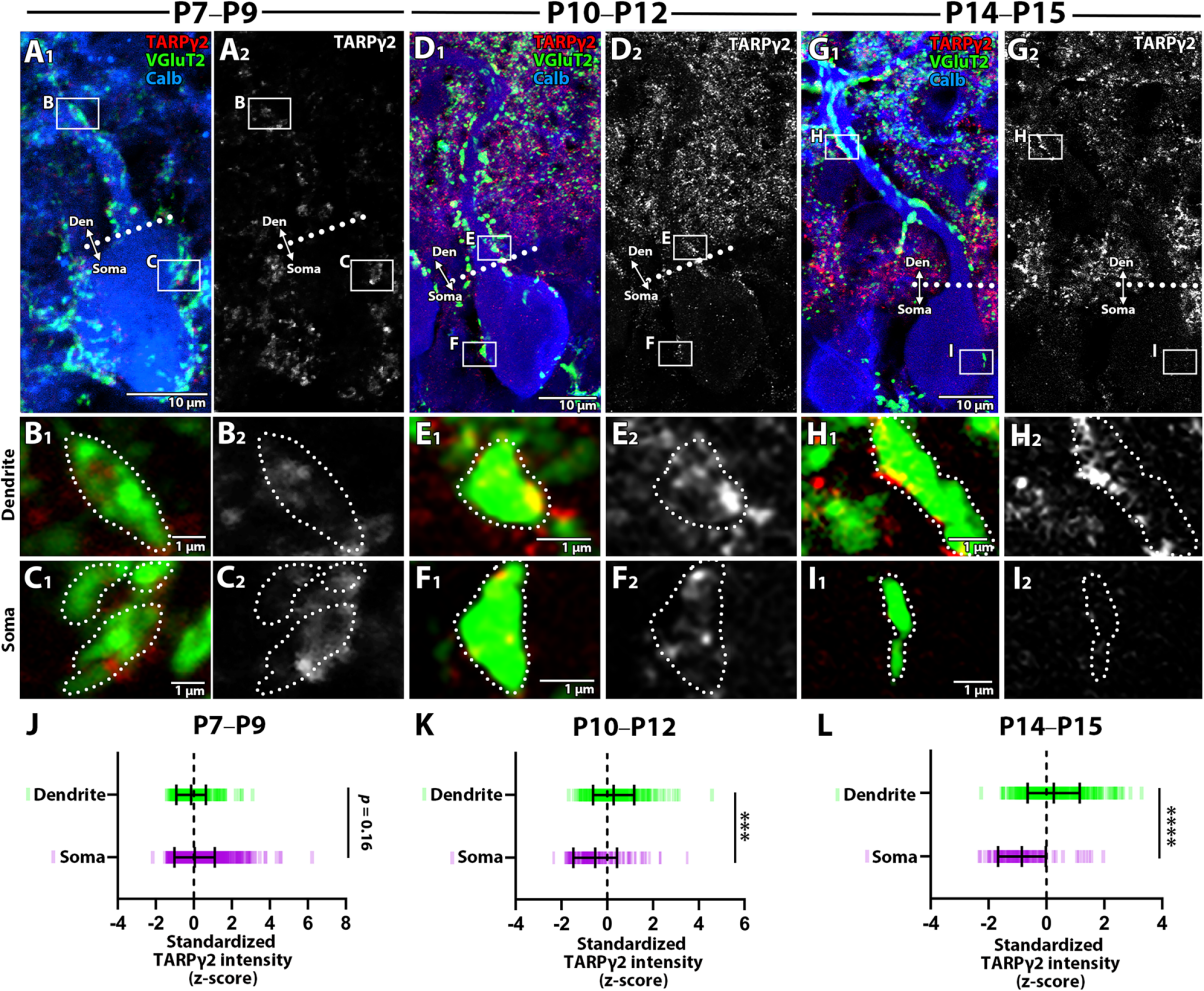
TARPγ2 expression across two classes of CF synapses at three developmental stages. ***A–I***, Triple fluorescent labeling for VGluT2 (green), TARPγ2(red), and calbindin (blue) to compare the expression level of TARPγ2 across the three types of CF synapses at P7–P9 (left), P10–P12 (middle), and P14–P15 (right). Dotted lines in ***A***, ***D***, and ***G*** represent the somato-dendritic border. Boxed regions in ***A***, ***D***, and ***G*** are enlarged in ***B***, ***C***, ***E***, ***F***, and ***H***, ***I***, respectively. Dotted lines ***B***, ***C***, ***E***, ***F***, ***H***, and ***I*** indicate the contour of CF terminals. ***J–L***, Summary plots comparing the standardized signal intensity of TARPγ2 in each class of CF synapses. Dashes represent the standardized signal in individual CF ROIs. The sample size used for statistical testing of synapses on dendrites and the soma is *n* = 270 and 772 CF ROIs from 5 PCs across 2 mice (P7–P9); *n* = 290 and 157 CF ROIs from 5 PCs across 2 mice (P10–P12); *n* = 380 and 114 CF ROIs from 5 PCs across 2 mice (P14–P15). Mean ± SD is shown for each plot. ***p < 0.001, ****p < 0.0001; Mann-Whitney *U* test.

Synaptic AMPAR expression depends on TARPγ2/stargazin (Chen et al., 2000; Schnell et al., 2002), and AMPAR-enriched synapses exhibit high TARPγ2 expression (Yamasaki et al., 2011; Yamasaki et al., 2016). To determine if dendritic CF synapse upregulation involves TARPγ2 expression, we performed triple immunofluorescence for calbindin (blue), VGluT2 (red), and TARPγ2 (red/white) (Fig. 7) and compared dendritic and somatic CF synapses. At P7–P9, TARPγ2 labeling was diffuse in both synapse types (Fig. 7*A–C*). By P10–P12, TARPγ2 labeling was intensified at dendritic CF synapses (Fig. 7*D*,*E*) but remained low at somatic CF synapses (Fig. 7*D*,*F*). At P14–P15, TARPγ2 labeling was further enhanced in dendritic synapses, while somatic synapse labeling decreased (Fig. 7*G–I*). Standardized signal intensity analysis showed comparable TARPγ2 labeling at P7–P9 (Fig. 7*J*; Mann-Whitney *U* test, *p* = 0.16). However, labeling became significantly more intense in dendritic synapses at P10–P12 (Fig. 7*K*; Mann-Whitney *U* test, *p* < 0.001) and P14–P15 (Fig. 7*L*; Mann-Whitney *U* test, *p* < 0.0001). These findings indicate that TARPγ2 expression is upregulated at dendritic “winner” CF synapses during and after the peak of dendritic translocation.

#### RIM1/2

**Figure 8.**
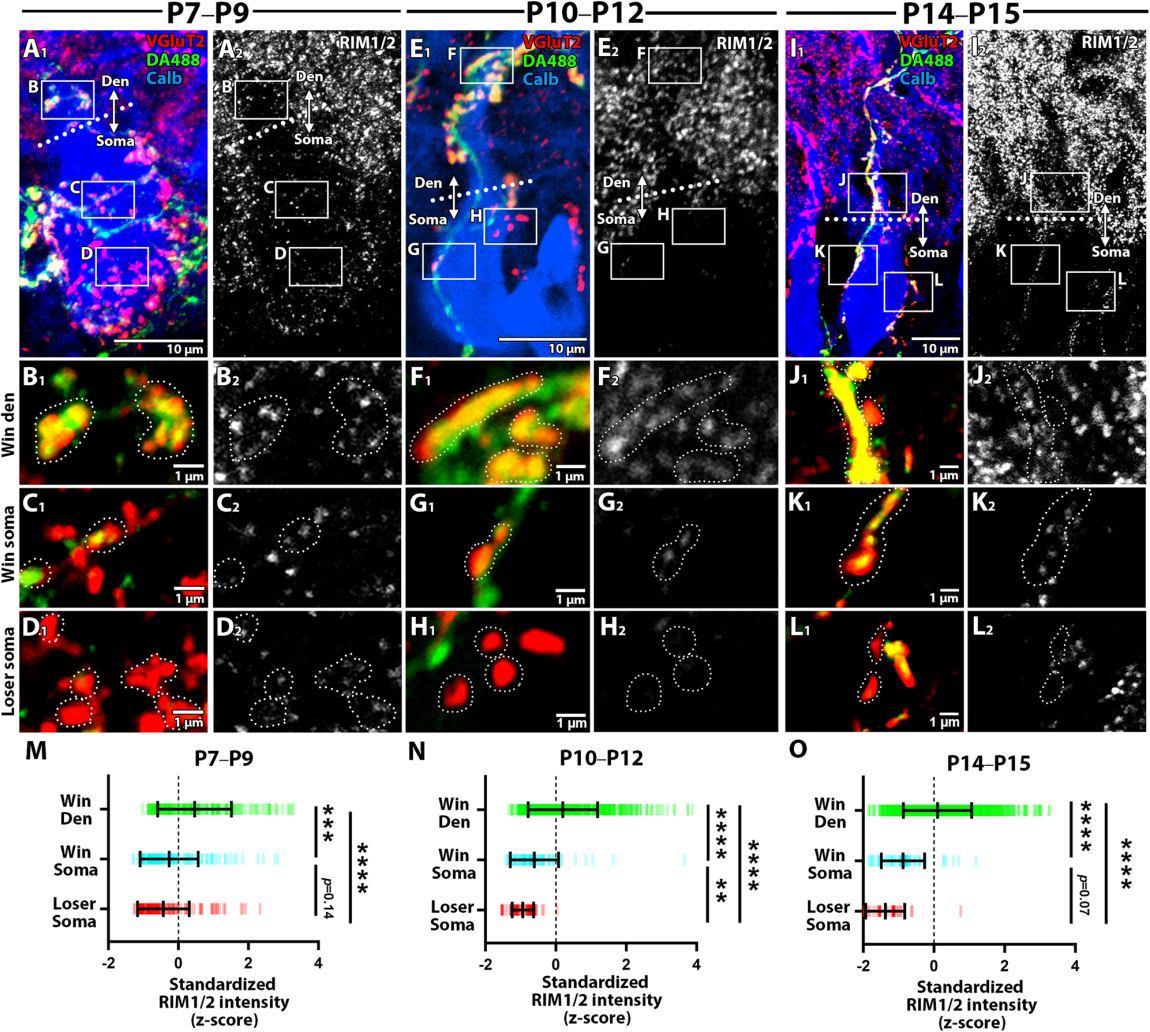
RIM1/2 expression across three classes of CF synapses at three developmental stages. Quadplex fluorescent labeling for DA488 (green), VGluT2 (red), calbindin (blue) and RIM1/2 (white) to compare the expression level of RIM1/2. See the explanation in Figure 5. The sample size used for statistical testing of “winner” dendrite, “winner” soma, and “loser” soma is *n* = 222, 189, and 171 CF ROIs from 8 PCs across 3 mice (P7–P9); *n* = 665, 93, and 80 CF ROIs from 10 PCs across 3 mice (P10–P12); *n* = 1,075, 64, and 38 CF ROIs from 8 PCs across 3 mice (P14–P15). Mean ± SD is shown for each plot. **p < 0.01, ***p < 0.001, ****p < 0.0001, Kruskal-Wallis test and post hoc Dunn’s multiple comparisons.

Presynaptic release function is another determinant of synaptic strength. Among various molecules involved, RIM plays a crucial role in organizing the presynaptic active zones for transmitter release (Kaeser and Regehr, 2014). We examined RIM1/2 expression by quadruple fluorescent labeling for calbindin (blue), VGluT2 (red), DA488 (green), and RIM1/2 (white) (Fig. 8). At P7–P9, intense punctate labeling for RIM1/2 was preferentially associated with the “winner” CF synapses in dendrites (Fig. 8*A*,*B*), whereas both “winner” and “loser” CF synapses in the soma showed diffuse and weak RIM1/2 labeling (Fig. 8*C*,*D*). This trend was further pronounced during and after the peak of dendritic translocation (Fig. 8*E–L*). As a result, RIM1/2 labeling was observed strongly at “winner” CF synapses in dendrites (Fig. 8*F*,*J*), weakly at “winner” synapses in the soma (Fig. 8*G*,*K*), and scarcely at “loser” synapses in the soma (Fig. 8*H*,*L*). The quantitative comparison showed that RIM1/2 labeling associated with “winner” CF synapses in dendrites was significantly higher than that of “winner” and “loser” CFs in the soma throughout the three stages (Fig. 8*M–O*; Kruskal-Wallis test followed by Dunn’s multiple comparisons tests, *p* < 0.001 for each). These results suggest that RIM1/2 is up-regulated only at the “winner” CF synapses translocated to dendrites.

## Discussion

During early postnatal development, redundant connections are eliminated, while necessary connections are strengthened to persist into adulthood. Strengthening a single CF is crucial for subsequent dendritic translocation and the establishment of CF mono-innervation (Kano et al., 2018). This study investigated the molecular and anatomical processes by which “winner” CF synapses are strengthened before, during, and after the peak of dendritic translocation.

A previous EM study on P3 and P7 mice revealed that potential “winner” and “loser” CFs are indistinguishable by the size of PSD (Wilson et al., 2019). Electrophysiological studies clarified that multiple CFs innervating each PC soma have similar synaptic strengths in neonates, and selective strengthening of a single “winner” CF occurs from P3 to P7 in mice (Hashimoto and Kano, 2003; Kawamura et al., 2013). These findings indicate that, prior to extensive dendritic translocation, CFs strengthen their innervation and compete with other CF inputs primarily by quantitatively increasing the number of synapses, which share similar structure and function. The current study revealed that at the peak of dendritic translocation (P10–P12), the total PSD area of dendritic and somatic spines synapsing with the “winner” CFs was significantly more extensive than that of spines associated with the “loser” CFs (Fig. 3*E*). Since the number of synapses per spine is comparable across the three classes of CF synapses, this area difference is primarily attributed to the difference in the individual PSD size at each synapse (Fig. 3*G*). Our present observations underscore that after P7, “winner” CFs further consolidate their innervation through qualitative changes, including structural differentiation of both pre- and postsynaptic elements (Figs. 2–4). These changes are accompanied by an upregulation of molecular determinants that enhance synaptic strength specifically at dendritic inputs, as evidenced by higher PSD95, AMPAR, TARPγ2 and RIM1/2 expression in dendritic “winner” CF synapses (Figs. 5–8). Our analysis highlights enhancements at dendritic synapses, but these observations could reflect upregulation at dendritic inputs, downregulation at somatic inputs, or a combination of both. This aspect warrants future investigation to clarify the underlying mechanisms.

Hebbian synaptic plasticity, such as long-term potentiation (LTP), is widely considered a basis for both functional and successive structural synapse strengthening (Matsuzaki et al., 2004; Holtmaat and Svoboda, 2009). In developing CF-PC synapses, LTP is presumed to promote the functional strengthening of a single “winner” CF. By the end of the first postnatal week, LTP can be elicited at CF-PC synapses by coincident CF activation and PC depolarization (Bosman et al., 2008; Ohtsuki and Hirano, 2008), which depends on a rise in cytosolic Ca^2+^ concentrations (Bosman et al., 2008). Dendrites have a higher density of voltage-gated Ca^2+^ channels (Usowicz et al., 1992; Llano et al., 1994) and a larger surface-to-volume ratio (Eilers et al., 1995) than the soma. Consequently, the “winner” synapses in dendrites likely experience more significant Ca^2+^ transients essential for LTP induction. This may result in an expansion and elaboration of the synaptic interface between axon terminals and dendritic spines, leading to an enlargement of the PSD area, a process associated with LTP induction (Desmond and Levy, 1988; Buchs and Muller, 1996; Cheetham et al., 2014). By contrast, at “loser” synapses, LTP is not induced (Bosman et al., 2008; Ohtsuki and Hirano, 2008), and the PSD size is reduced or even fragmented, as we showed in the present (Figs. 3*F*,*G* and 4*H*,*I*) and a previous study (Ichikawa et al., 2011). Despite differences in total and individual PSD area (Figs. 3*F*,*G* and 4*H*,*I*), spine volumes remained comparable across all three CF synapse classes (Figs. 3*E* and 4*G*). The differential PSD-spine volume correlations between “loser” and “winner” synapses suggest distinct plasticity directions, protein stoichiometry, and composition (Borczyk et al., 2019). The anatomical features of “winner” CF synapses (Figs. 2–4) indicate that these synapses have undergone activity-dependent alterations, further ensuring their high synaptic strength and dominance over “loser” CF synapses.

During and after the peak of dendritic translocation, “winner” CFs that translocated to dendrites were associated with more postsynaptic AMPARs compared to “winner” and “loser” CF terminals in the soma (Fig. 6*N*,*O*). Given that the amplitude of postsynaptic responses attenuates with the distance from the soma due to dendritic filtering (Hashimoto et al., 2001; Roth and Häusser, 2001), the preferential up-regulation of AMPARs at dendritic “winner” CF synapses would serve to compensate for and overcome the distance from the soma and to maintain the synaptic efficacy. Furthermore, before the peak of translocation, “winner” CF synapses in the soma were associated with more AMPARs than “loser” synapses in the soma (Fig. 6*A*). This is consistent with a previous electrophysiological finding that the differentiation into “winner” and “loser” CFs occurs at the soma before the stage of extensive dendritic translocation (Hashimoto et al., 2009a). Before the extensive dendritic translocation, differences in presynaptic release function across multiple CF inputs are less distinct (Hashimoto and Kano, 2003); instead, postsynaptic mechanisms seem to make the difference among multiple CF inputs. For instance, CF-LTP selectively strengthens “winner” CF synapses via postsynaptic mechanisms (Bosman et al., 2008). Indeed, higher AMPAR expression at “winner” CF synapses was accompanied by the incorporation or upregulation of PSD95 (Fig. 5) and TARPγ2 (Fig. 7). Notably, TARPγ2 regulates synaptic AMPA receptor expression and function (Yamazaki et al., 2010; Kawata et al., 2014; Yamasaki et al., 2016) and its deficiency weakens “winner” CF strength (Yamazaki et al., 2010; Kawata et al., 2014) and disrupts the dendritic translocation of “winner” CFs (Kawata et al., 2014). Thus, investigating the role of TARPγ2/stargazin and the additional factors that regulate its expression would provide valuable insights into the postsynaptic mechanisms underlying activity-dependent synaptic strengthening.

On the presynaptic side, the “winner” CF terminals translocated to dendrites were abundant in RIM1/2 (Fig. 8). RIM proteins recruit Ca^2+^ channels to the presynaptic active zone and modulate Ca^2+^-channel opening times, thereby facilitating the transmitter release (Kiyonaka et al., 2007; Kaeser et al., 2011; Kaeser and Regehr, 2014). Maturation of “winner” CF synapses is marked by increased release probability and multivesicular release occurring after dendritic translocation (Hashimoto and Kano, 2003). The increase in release probability is reportedly mediated by retrograde signals such as progranulin, which is released from PCs and activates Sort1 on CF terminals, in the second postnatal week (Uesaka et al., 2018). The selective up-regulation of RIM1/2 at “winner” CF synapses may play a critical role in the maturation of release function, thereby facilitating the presynaptic mechanism for activity-dependent synaptic strengthening. Since CF release function is maintained by postsynaptic AMPAR activity (Kakizawa et al., 2005), the abundance of AMPARs in the postsynapse and RIMs in the presynapse likely creates a positive feedback loop that strengthens “winner” CFs, facilitating dendritic translocation and CF mono-innervation.

Our previous EM study demonstrated that the density of somatic CF synapses peaked at P9, when pericellular nests around PC somata develop and CF–PC synapses account for 88.1% of the total somatic synapses (Ichikawa et al., 2011). This high fraction drastically declines to 5.8% at P15% and 0.5% at P20. Although “winner” CF synapses in dendrites and the soma share similar structural characteristics (Figs. 2 and 3), somatic “winner” synapses had significantly lower PSD95, AMPAR, and RIM1/2 expression levels after P10, suggesting their weaker synaptic strength than dendritic “winner” synapses (Figs. 5–8). Given that “loser” CFs are eliminated within a few days after the relative strength difference exceeds a certain threshold (Hashimoto and Kano, 2003), weak “winner” CF synapses in the soma would be eliminated eventually. Changes in the PSD95 levels across the three classes of CF synapses only became evident at later stages (Fig. 5) may, at least partly, reflect the slow turnover rate of PSD95 (Kuriu et al., 2006). Moreover, it has been shown that, even after CF terminals dissociate from the soma, somatic spines remain and retain PSD95 expression transiently until somatic synapses are replaced with inhibitory basket cell synapses (Ichikawa et al., 2011). In parallel, PF synapse formation is accelerated on PC dendrites (Altman and Bayer, 1997). Importantly, GABAergic inhibition by basket cell-PC synapses and mGluR1 activation at PF-PC synapses both promote CF synapse elimination from the soma (Uesaka et al., 2014; Nakayama et al., 2024). Thus, massive elimination of somatic CF synapses likely begins with functional weakening by down-regulation of AMPARs and is followed by complete degradation of PSD95-containing scaffolds, which process is fueled heterosynaptically by GABAergic inhibition and mGluR1 activation.

In conclusion, our study demonstrates the selective molecular and anatomical strengthening of CF synapses that have translocated to dendrites, granting them an irreversible competitive advantage over somatic CF synapses.
